# Automated identification of borrowings in multilingual wordlists

**DOI:** 10.12688/openreseurope.13843.2

**Published:** 2021-08-24

**Authors:** Johann-Mattis List, Robert Forkel

**Affiliations:** 1 Department of Linguistic and Cultural Evolution, Max Planck Institute for Evolutionary Anthropology, Leipzig, Thüringen, 04103, Germany

**Keywords:** computational linguistics, historical linguistics, lexical borrowing, borrowing detection, computational historical linguistics

## Abstract

Although lexical borrowing is an important aspect of language evolution, there have been few attempts to automate the identification of borrowings in lexical datasets. Moreover, none of the solutions which have been proposed so far identify borrowings across multiple languages. This study proposes a new method for the task and tests it on a newly compiled large comparative dataset of 48 South-East Asian languages from Southern China. The method yields very promising results, while it is conceptually straightforward and easy to apply. This makes the approach a perfect candidate for computer-assisted exploratory studies on lexical borrowing in contact areas.

## Plain language summary

Lexical borrowing, the transfer of words from one language to another, is one of the most prominent aspects of language evolution. Despite its prominence, only a few attempts have been made to create computational methods that would identify borrowings automatically from linguistic datasets. In this study, we propose a new method which is straightforward and easy to apply. We test it on a dataset of 48 languages from Southern China and find that it yields good results. We conclude that the method may be useful for future computer-assisted studies on lexical borrowing.

## Introduction

Few phenomena in linguistics are as pervasive as language contact ( Onysko, 2019). It is the first factor that needs to be excluded when searching for genealogical language relationships or universals in the languages of the world. It is an indispensable aspect of studies on human cognition, since any study trying to explain the human language faculty must explain how humans can master a multitude of languages. Language contact is so widespread that it was the first factor of language change identified by early philosophers (compare Plato's *Kratylos* dialogue), more than two millennia before scholars began to understand that all languages are subject to change even without contact ( Schleicher, 1863). Due to its pervasiveness, language contact is also a powerful witness to human prehistory, as illustrated by phonetically similar names for the sweet potato in Polynesian and Quechuan languages, which provide evidence that its transfer to Polynesia was due to human contact ( Montenegro *et al.*, 2008).

While comparative linguistics has experienced a quantitative turn during the past decades, studies on language contact are still almost exclusively carried out manually, and quantitative studies of language contact phenomena are still in their infancy ( List, 2019a). This also applies to automated methods for the identification of lexical borrowings. Although some methods have been proposed, none of them deal with *multilingual wordlists*. In this study, we propose a new method for this task and test it on a newly compiled dataset of South-East Asian languages. The method yields very promising results, while it is conceptually straightforward and easy to apply. This makes the approach a perfect candidate for computer-assisted exploratory studies on lexical borrowing in contact areas.

## Background

Since historical linguists typically try to exclude borrowings from their analysis rather than making borrowings part of their analysis, methods for the identification of language contact situations have never really left the "shortcut status", where scholars make use of a certain number of ad-hoc criteria to discuss the degree of language contact in a certain region. It is therefore not surprising that computational methods for the identification of borrowed traits are still in their infancy ( List, 2019a), although computational methods in historical linguistics have been flourishing in the past decades. Of the few methods which have been proposed so far, there are phylogenetic network approaches which do not require a strict tree-like phylogeny to model language evolution, but instead assume that certain traits can also be transferred laterally through contact ( List, 2015; List *et al.*, 2014a; List *et al.*, 2014b; Nakhleh *et al.*, 2005; Nelson-Sathi *et al.*, 2011). While most phylogenetic network approaches deal with lexical data and try to infer lexical borrowings, recent studies have shown that these approaches can likewise be used to study the areal spread of grammatical traits ( Cathcart *et al.*, 2018).

On the other hand, scholars have tried to identify borrowings directly with techniques for automated sequence comparison. These methods treat phonetically transcribed words in spoken languages as sound sequences and then seek to identify similar sequences by using techniques originally designed for computer science and evolutionary biology ( List, 2014). Since sequence comparison techniques are primarily applied to identify cognate words (words shared by common inheritance), most methods that make use of them can only identify borrowings between genetically unrelated languages ( van der Ark *et al.*, 2007; Mennecier *et al.*, 2016; Zhang *et al.*, 2021) and only a few attempts have been made to identify borrowings in genetically related languages ( Hantgan & List, forthcoming).

## Materials and methods

### Materials

For this study, a new dataset was compiled by aggregating several existing datasets on South-East Asian languages spoken in Southern China. The core of the dataset is a collection of 25 Hmong-Mien language varieties documented by Chén (2012). This dataset was standardized in an earlier study ( Wu *et al.*, 2020) by converting it to the standard formats recommended by the Cross-Lingustic Data Formats initiative (CLDF, Forkel *et al.*, 2018). Using the CLDFBench toolkit ( Forkel & List, 2020) allows regular and transparent data conversion to CLDF including links to reference catalogs, such as Glottolog for language varieties ( Hammarström *et al.*, 2021) and Concepticon for concepts ( List *et al.*, 2021a). In addition, CLDF makes transcriptions transparent by linking segments to the B(road)IPA transcription system, which is a stricter version of the standard International Phonetic Alphabet ( IPA, 1999), for the representation of speech sounds ( Anderson *et al.*, 2018; List *et al.*, 2021b).

Having shown earlier that CLDF greatly facilitates the aggregation of data from diverse sources ( List *et al.*, 2018), we assembled data on additional South-East Asian language varieties from sources which were either already converted to CLDF in earlier works ( Běijīng Dàxué, 1964) or prepared specifically for this study ( Wang, 2004). Table 1 shows the eight core datasets that were used in this study. In addition, Chinese dialect data is available in the form of lists of character pronunciations. While these do not provide any strict information on actual words, since individual characters correspond to morphemes in Chinese, it can still be useful to include them in larger multilingual collections, since they may fill gaps where the available data on words in Chinese dialects is sparse. For this reason, the character readings from two datasets ( Běijīng Dàxué, 1962; Hóu, 2004) were roughly linked to common concepts in our base datasets in order to increase the coverage for individual language varieties.

**Table 1.** Sources of the data selected for this study. Note that due to the rather low coverage of data without base list of 250 concepts in some datasets, several varieties were aggregated from two or more sources. This is indicated in the CLDF version of the dataset. For character readings, where rudimentary Concepticon mapping was carried out on a very selective basis, only the number of concepts linked to Concepticon is shown in the Source column, also indicated by an asterisk.

ID	Source	Family	Varieties		Concepts	
			Source	Selected	Source	Selected
beidasinitic	Běijīng Dàxué (1964)	Chinese dialects	18	6	905	146
beidazihui	Běijīng Dàxué (1962)	Chinese dialects (characters)	19	4	*518	171
castrosui	Castro & Pan (2015)	Sui dialects (Tai-Kadai)	16	3	608	211
castroyi	Castro *et al*. (2010)	Loloish dialects (Sino-Tibetan)	6	1	540	222
castrozhuang	Castro & Hansen (2010)	Zhuang dialects (Tai-Kadai)	20	8	511	243
chenhmongmien	Chén (2012)	Hmong-Mien	25	23	888	250
housinitic	Hóu (2004)	Chinese dialects	40	10	180	61
houzihui	Hóu (2004)	Chinese dialects (characters)	40	9	*155	77
liusinitic	Liú *et al*. (2007)	Chinese dialects	19	5	201	130
wangbai	Wang (2004)	Bai dialects (Sino-Tibetan)	9	1	471	144

**Figure 1.**
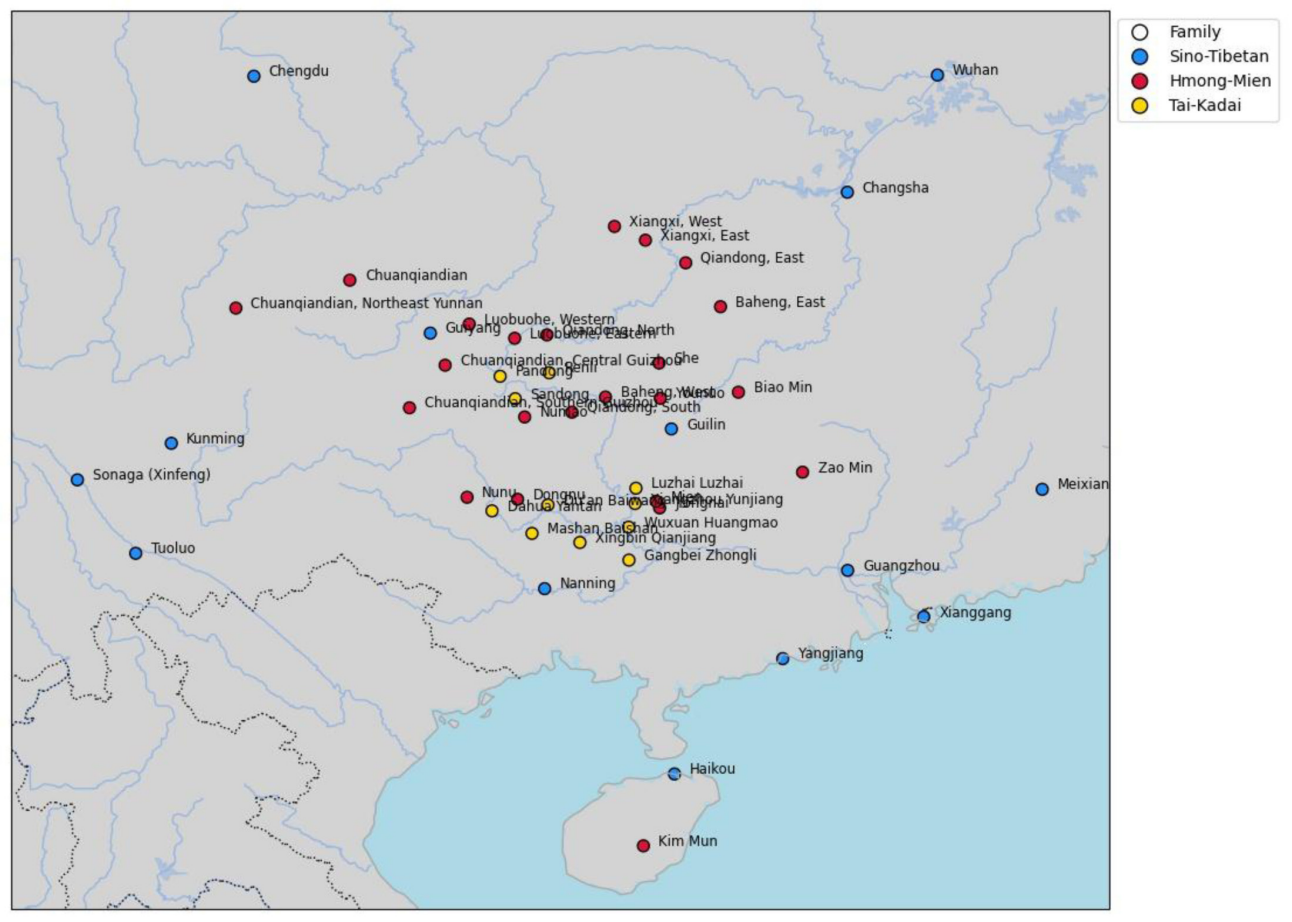
Languages in the sample.

From these datasets, a subset of 48 language varieties (23 Hmong-Mien languages, 11 Tai-Kadai languages, and 14 Sino-Tibetan languages) and 250 concepts were selected. The criterion for data and concept selection was the general coverage of individual language varieties with respect to the 250 concepts chosen, and the geographic proximity of the languages in the sample. While the coverage with respect to the concepts for which there is a word form in individual language varieties is reasonably high in most cases, there are some outliers, mostly from Chinese dialects, in which more than 40% of the concepts are missing. While a low coverage for certain varieties would be problematic for phylogenetic studies ( Sagart *et al.*, 2019), it was nevertheless decided to keep these language varieties in the sample, mostly because their geographic position makes them interesting candidates for donor and recipient languages in the current sample. Figure 1 shows the geographic distribution of the languages in our sample.

While it would be desirable to extend the data further, we consider the current collection as sufficient for initial experiments on automated borrowing detection. Extending the dialect data, specifically for the Chinese dialects, could in theory be achieved in the future by integrating information on individual dialects extracted from dialect dictionaries, such as the collection of Lǐ (2002), which comprises dialect dictionaries for 42 dialect varieties. The integration of this and similar sources, however, cannot be done in a straightforward way, since the data was usually not collected from established questionnaires, but is instead listed only by dialect headwords (given by Chinese characters with their pronunciations), which are furthermore only sporadically translated (see Kurpaska, 2010: 128–183 on the structure of the dictionary collection by Lǐ, 2002). As a result, finding the counterpart for a concept like “sun” requires to search actively for a word that is potentially cognate with “sun” in the target dialect in a first step, and to verify this entry in a second step with the translation provided in the resource. Thus, although it would be desirable to improve on our current data basis, this endeavour would largely exceed the scope of the current study.

### Methods

***Automated borrowing detection.*** The new method for the identification of borrowings in multilingual wordlists is based on a very straightforward workflow that proceeds in two steps. In the first step, traditional methods for cognate detection are used to identify *language-family-internal cognate sets* in the data. In the second step, all language-internal cognate sets are compared across language families and clustered into sets of *potentially borrowed words* once the overall average distance among the cognate sets is below a certain threshold. For the first step, it is useful to use a conservative cognate detection method which searches for deep genealogical similarities among word forms. An example for such a method is the LexStat algorithm for automated cognate detection, which has been proposed earlier (see List, 2012a and List *et al.*, 2017), or its modification, which searches for partial cognates (words which are not entirely cognate but only in parts of their individual morphemes) instead of full cognates ( List *et al.*, 2016). For the second step, it is useful to employ a less conservative method for cognate detection that searches for superficial phonetic similarities rather than deep similarities based on regular sound correspondences. Here, the sound-class-based alignment (SCA) method for pairwise and multiple phonetic alignment ( List, 2012b) is a good candidate, specifically also because studies on pairwise borrowing detection have shown that SCA outperforms edit distance in this task ( Zhang *et al.*, 2021).

For our specific use case, we decided to use the LexStat algorithm adjusted for partial cognate detection ( List *et al.*, 2016), since it is well known that South-East Asian languages show frequent compounding patterns which cannot be captured when searching for full cognates in the data (for a detailed discussion on partial cognacy, we refer interested readers to Hill & List, 2017). The LexStat Partial algorithm expects words to be segmented into morphemes by the user and then uses a network approach to cluster morphemes which ocurr in the same concept slot of a given wordlist into sets of cognate morphemes, corresponding to partially cognate words. Since one would expect, however, that borrowings involve full words, it seems useful to employ a full word alignment algorithm for the second stage. When searching for partial cognates in a first instance, this means that one needs to find a way to convert partial cognates to full cognates later. Since words can share cognate morphemes in different parts, the partial cognate relation is not transitive (word *A* with form *xyz* can be partially cognate to word *B* with form *x* and to word *C* with form *ya*, while word *B* is not partially cognate with word *C*, but a word D with form *ab* would be cognate with *D*), a specific conversion procedure that transitivizes cognate relations is needed. For this purpose, we decided to use a new method that we recently developed ( Wu & List, 2021). This method is based on a greedy algorithm that assigns partial cognates to full cognate sets which have at least one cognate set in common. This method starts from the most frequently recurring partial cognate, assigning all words which contain this morpheme to the same cognate sets, and then proceeds with the remaining word forms which have not yet been assigned to a cognate set until all words have been visited (yielding the merger of *A* with *B* due to their shared cognate set *x* and then proceed to cluster *C* and *D* due to their shared cognate *a*).

In order to compare two cognate sets expressing the same meaning from two different language families, the new method proposed here first computes pairwise SCA distances for each possible word pair assembled from languages from different language families. The distances are all stored in memory and then averaged. If the average distance is lower than a user-defined threshold, a link between the cognate sets is drawn. After all cognate sets from different languages have been compared in this fashion, the method searches for all *connected components* in this cognate set network and assigns all cognate sets appearing in the same connected component to the same set of potentially borrowed words.

This method does not resolve the direction of borrowings. But instead of earlier approaches, which only identify pairs of potentially borrowed words, it allows to cluster words into *xenologs*, that is, sets of words which are not entirely related by common descent, but also by lateral transfer ( List, 2016). Furthermore, since all our methods needs as evidence is to find that two words are attested in different language families (following the idea first expressed in van der Ark *et al.*, 2007), the method might even detect borrowings from a third language family, with the original word (or cognate forms thereof) not being reflected in the sample.

**Figure 2.**
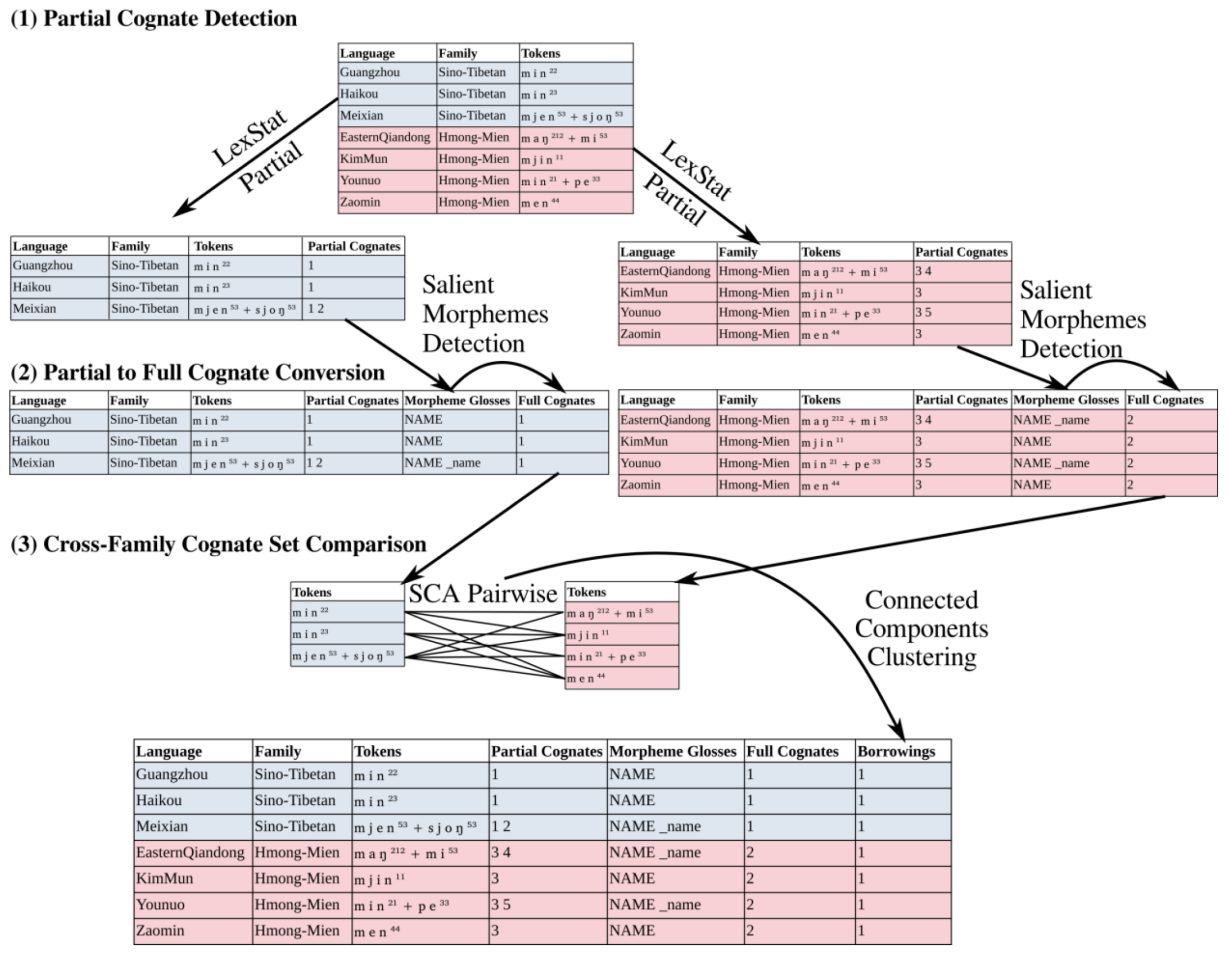
Workflow example.

Figure 2 shows a rudimentary workflow example in which the major steps are displayed. In step (1), partial cognates are inferred with the help of the LexStat-Partial algorithm, for each language family in separation. In step (2) the greedy algorithm by Wu & List (2021) is used to convert partial to full cognates based on the identification of those cognate morphemes which recur in the largest part of the data. In step (3), individual cognate sets across language families are compared with each other using the SCA algorithm for pairwise alignments. Based on these scores (not shown in the figure), a network of cognate sets is constructed in which cognate sets are connected which show an average distance score beyond a user-defined threshold.

The selection of thresholds is important for the identification of partial cognates and for the identification of cross-language-family cognate sets (or sets of xenologous words). Since both thresholds are crucially intertwined with each other, it is not easy to come up with an objective solution for threshold detection. Increasing the threshold for cognate detection, for example, will cluster more words into the same cognate set in related languages. The larger the language-internal cognate sets, however, the higher the chance that borrowing sare rejected if the second threshold is considerably low. Since threshold detection in cognate detection tasks is a problem that has no straightforward solution by now, all we can do for now is to derive thresholds by means of trial and error. While this may not seem optimal, we emphasize that we consider the major value of our approach not in the detection of indisputible truths regarding cognate and borrowed words, but rather as a tool supposed to aid linguists in their research, which would necessarily invole a manual refinement of automatic analyses.

An alternative way to achieve results similar to our workflow would be to use the well-established cognate detection algorithms on the whole dataset without splitting the data into language families first, and then infer potential borrowings by identifying those cognate sets which appear in more than one language family. While this approach has been used successfully in a computer-assisted study in the past ( Hantgan *et al.*, 2020), our approach has several advantages, as it allows us not only to combine different methods for language-family-internal and language-family-external cognate detection, but also runs faster, since a much smaller part of the data has to be compared in a one-to-one fashion. Since the implementation of this alternative method for borrowing detection can be done in a straightforward way with the help of the LingPy library, we added the code to the code package accompanying this study. 

***Annotation of borrowings in multilingual wordlists.*** In order to allow us to test the new method against human judgments, the data was annotated manually, using the EDICTOR ( List, 2017), a web-based tool for curation of etymological data in historical linguistics. The data was annotated in two stages. First, cognate sets were identified inside all language families in our sample. In a second stage, cognate sets were themselves assembled into larger sets of potentially borrowed words.

**Figure 3.**
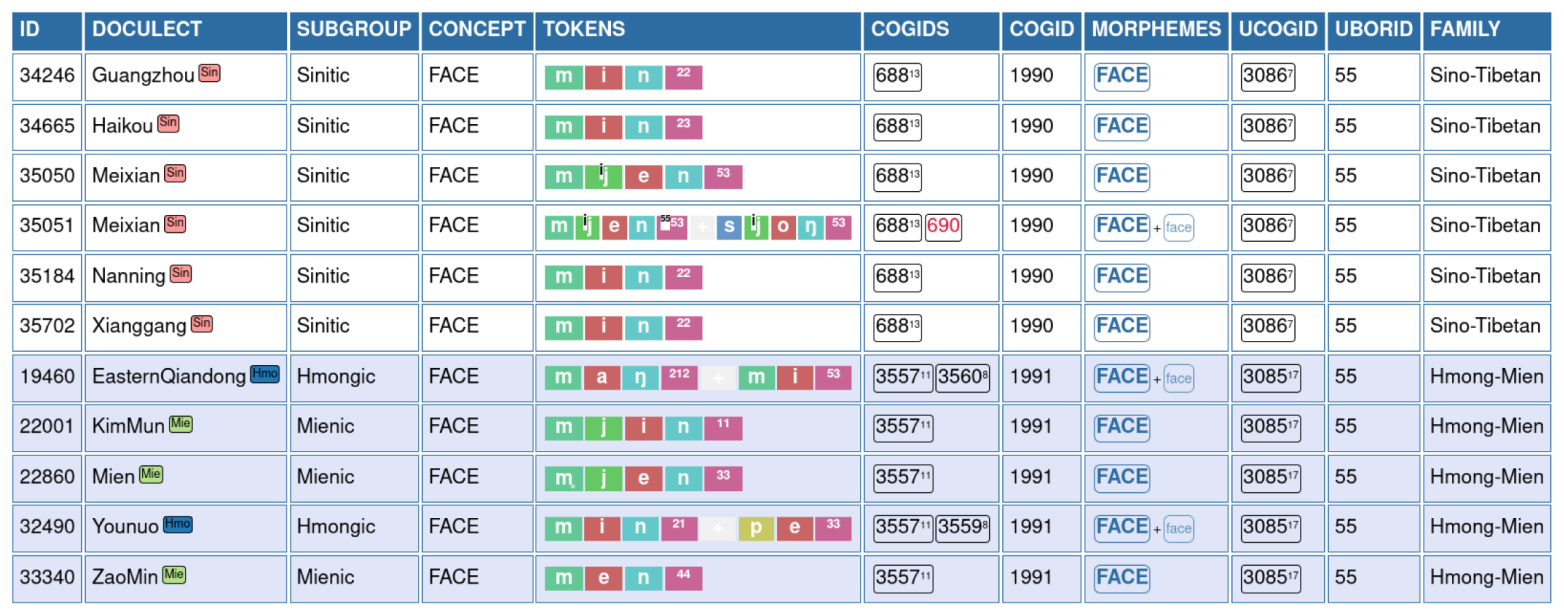
EDICTOR annotation of borrowings.

An example for this annotation is shown in Figure 3, where words for "face" in Hmong-Mien and Sino-Tibetan are compared with each other. The table shows our annotation procedure and how data are displayed in the EDICTOR application The ID is used to refer to the original data point and allows us to trace the data from the original sources and across different files. The DOCULECT column provides a language identifier, which also provides rudimentary subgroup information (also displayed in the column SUBGROUP), which was made available with the most recent version of EDICTOR. The column TOKENS shows the sound sequences in segmented, normalized form, segmenting the individual sounds (which may be transcribed by more than one characters) by a space and uses the symbol + as an additional character to indicate morpheme boundaries. Partial cognate identifiers are manually added to the data in the column COGIDS, which are provided on a language-family-internal basis only and indicate with the help of unique identifiers which morphemes of a word form a group of xenologous words. The column MORPHEMES indicates which parts of the word express the main (or salient aspect of the) meaning "FACE" and help us to convert the partial cognates into full cognates, following Wu & List (2021) in taking the salient (or main) morphemes as the criterion to convert partial to full cognates. Column UBORID provides the user judgment on potentially borrowed words. Where known, the SOURCE of a borrowing was also indicated, as illustrated for the FACE by 面, Middle Chinese *mjienH* ( Baxter, 1992). Additionally, information on the language family, to which a language belongs, is displayed in the column FAMILY, which is routinely provided in the CLDF datasets, from which the data was aggregated. 

***Data Formats and Data Representation.*** Having been collected from datasets originally provided in the unifying CLDF formats, which standardizes elicitation glosses for concepts via Concepticon ( List *et al.*, 2021a), language names via Glottolog ( Hammarström *et al.*, 2021), and phonetic transcriptions via CLTS ( List *et al.*, 2021b), the aggregated dataset along with our manual and automated analyses is also provided in the form of a CLDF dataset, which is curated on GitHub ( https://github.com/lexibank/seabor) and archived with Zenodo (Version 1.0, https://doi.org/10.5281/zenodo.5037101). For a detailed introduction of the tabular CLDF formats, we refer the readers to Forkel *et al.* (2018).

As mentioned in the previous section, the annotation of partial cognates, full cognates, and borrowing candidates is carried out with the help of the EDICTOR tool. The format underlying this tool is based on a rather well-established tabular representation of lexical data in which each row of a table is reserved for one word form in a particular language and columns provide individual data on each word form. The EDICTOR database is curated online and can be directly accessed in read-only form by interested scholars ( https://digling.org/links/seabor.html). For more information on the format, we refer interested readers to an initial study by Hill & List (2017), where partial cognacy and morpheme glosses were first discussed in detail, as well as the follow-up studies by Schweikhard & List (2020), where these formats were further extended, and Wu & List (2021), where a detailed discussion of the problem of partial cognacy is given.

***Implementation.*** The new method was implemented as part of the LingRex Python package ( List & Forkel, 2021a, Version 1.2). LingRex is an extension of LingPy which offers code that is specifically useful for the detection of sound correspondence patterns ( List, 2019b) and the prediction of words which have not been elicited from cognate sets ( Bodt & List, 2021). With this study, LingRex was further extended by the new method for the identification of borrowed words. The methods used for the efficient manual annotation of borrowed words were introduced as part of the most recent version (2.0.0) of the EDICTOR tool ( List, 2021). Plots made in this study were carried out with the help of CLDFViz ( Forkel, 2021), a Python package which facilitates the static and interactive visualization of data provided in CLDF, which was expanded to allow for the specific requirements for this study. The supplementary material accompanying this study offers both the data and the code needed to replicate the experiments which are reported in this study. Users who wish to replicate the study reported here can find all necessary information in the file “workflow.md”, where all steps of the workflow (including the creation of figures) are explained.

## Results

### General results

In order to test the new method for the identification of borrowings in multilingual datasets, the data was analyzed by using the default settings of the partial cognate detection algorithm (threshold of 0.50 and 10000 iterations in the permutation test) in order to search for language-family-internal cognates. We then ran the cross-language-family cognate detection method in order to identify potential borrowings, using a threshold of 0.3 in this step, since this threshold was the optimal one reported for the test of the SCA method for pairwise borrowing detection reported by Zhang *et al.* (2021).

As a first test of the methods, we compared the manually annotated cognates and borrowings with the cognates and borrowings which the automated method identifies, using the B-cubed scores ( Amigó *et al.*, 2009) as a measure to compare automated with manual cognate and borrowing judgments in terms of precision, recall, and F-scores. B-cubed scores are a technique for the comparison of two partions of the same data for similarity, and range between 1 and 0. A precision of 1 means that only items which also belong to the same partition in the gold standard are assigned to the same partition in the test, which is identical with no false positive decisions by the algorithm. A recall of 1 means that all items which were assigned to the same partition in the gold standard are also assigned to the same partition in the test, which is identical with no false negative decisions by the algorithm. Combining both scores by taking their harmonic mean, yields the F-score, which has become a standard way to compare cognate detection methods in historical linguistics by now (see List, 2014: 189 for details on the computation).

Despite the intensive degree of contact among South-East Asian languages spoken in Southern China, the number of sets of xenologous words in our data is still considerably small, resulting in sparse partitions in which only a small number of words is assigned to larger clusters. In order to compare how well our algorithms perform in borrowing detection, it is therefore important to test the methods against a base line. Here, we follow an idea first expressed in List (2019c) to contrast the results with a lumper and a splitter as a baseline. The lumper assigns all words to the same cognate set, no matter how similar or how dissimilar they are. The splitter assigns all words to different cognate sets. Both baselines help us to put our individual cognate and borrowing detection results into context.

The results of this first experiment show that the method works sufficiently well, as shown in Table 2, reaching F-scores of 0.88 for the automated cognate detection task, and 0.87 for the automated borrowing detection task. The method outperforms the lumper and splitter baselines. The lumper baseline which proves to be most efficient for the cognate detection task achieves 0.72, which is in strong contrast to the F-scores reached by our automated cognate detection method, and the splitter, which proves to be best on the borrowing detection, where the majority of partitions are singletons, because words cannot be shown to be in a xenologous relation with any other words, receives F-scores of 0.8. That the difference between the splitter baseline and our method is only 0.07 calls for specific attention, since it shows that the fact that words which cannot be assigned to borrowings are assigned to different cognate sets make up for a large part of the high scores, and we can see that the B-cubed scores should never be taken at face value but always compared to a baseline. 

**Table 2.** Results for the evaluation of the automated workflow compared to the gold standard.

Method	Precision	Recall	F-score
automated cognate detection	0.90	0.87	0.88
automated borrowing detection	0.94	0.81	0.87
lumper baseline for cognate detection	0.57	1.0	0.72
lumper baseline for borrowing detection	0.19	1.0	0.32
splitter baseline for cognate detection	1.0	0.25	0.39
splitter baseline for borrowing detection	1.0	0.66	0.80

Nevertheless, although these results should be taken with a certain care, since no further test sets are available, and no proper division into test and training data has been carried out, the scores can be considered sufficient to prove that the method proposed here is basically useful when searching for words shared across different language families.

In order to explore whether our approach of using two different methods (one “deep” and one “shallow” approach) to identify cognates on the one hand and borrowings on the other hand, we ran a couple of experiments with varying thresholds using the alternative approach to borrowing detection proposed by Hantgan *et al.* (2020) mentioned above. The approach by Hantgan *et al.* (2020) simply compares all words expressing the same concept in all languages with each other and searches for cognate words, using the SCA method for automated cognate detection ( List, 2012a) and later identifies those cognate sets as potentially influenced by borrowing which recur across more than one language family. In contrast to our two-step procedure, this method requires only one threshold. In order to test to which degree the choice of the threshold influences the F-scores achieved when searching for family-internal cognates as opposed to searching for cross-family xenologs, we ran the partial and the full cognate detection method with shallow SCA distances and with deep LexStat distances derived from automatically inferred sound correspondences for consecutive thresholds ranging from 0 to 1 in steps of 0.05.

Table 3 shows the thresholds which yield the best scores for the cognate and the xenolog detection task in the shallow (SCA) variant and the deep LexStat variant for partial and full cognates. As can be seen from these results, the two LexStat variants perform better in the cognate detection task. The partial variants outperform their respective non-partial counterparts. In the xenolog detection task, however, the SCA proper approach performs almost as well as the LexStat proper approach (0.85 vs. 0.86), and the variants which search for full as opposed to partial cognates outperform their respective counterparts.

**Table 3.** Results for the evaluation of different settings for approaches using a single threshold.

Method	Threshold	Cognates	Xenologs
SCA-Partial	0.35	0.86	0.73
SCA-Partial	0.15	0.76	0.81
SCA	0.40	0.84	0.74
SCA	0.15	0.74	0.85
LexStat-Partial	0.55	0.89	0.75
LexStat-Partial	0.40	0.83	0.83
LexStat	0.6	0.87	0.81
LexStat	0.5	0.84	0.86

**Figure 4.**
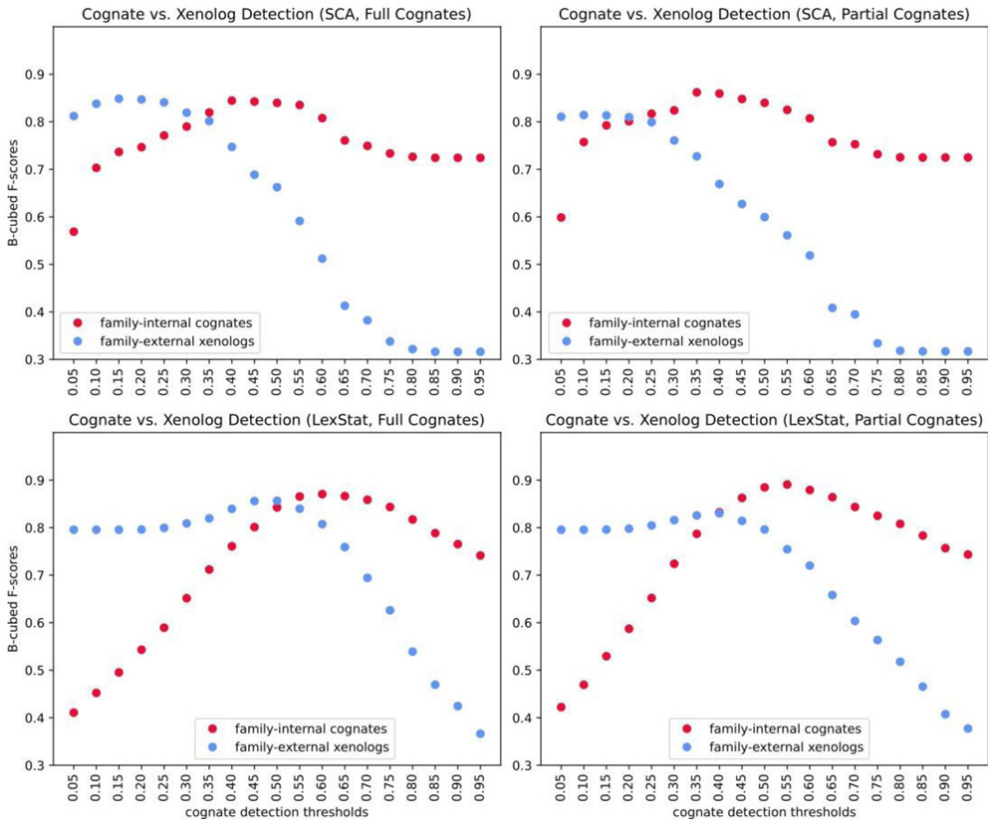
Comparing different settings and thresholds.

Figure 4 shows plots of the four analyses, contrasting the B-cubed F-scores achieved for the two different tasks. As can be seen from these plots, all approaches reach the best F-scores for different thresholds, depending on the task and the cognate detection task requires larger thresholds than the borrowing detection tasks in all cases. This shows that our approach of splitting the borrowing detection enterprise into two distinct tasks, one that seeks to detect language-family internal cognates and one which compares these cognate sets to find potential borrowing candidates, is generally justified.

### Specific results

Assuming that the method works well enough to capture at least recent borrowing events in our dataset on South-East Asian languages in Southern China, it is interesting to check whether these borrowings reflect specific patterns. In the linguistic literature it has, for example, for a long time been assumed that certain words are more resistant to borrowing than other words, mostly due to the meanings they express ( Swadesh, 1952; Swadesh, 1955). Given that the dataset was assembled from individual CLDF datasets which are themselves linked to the Concepticon project ( List *et al.*, 2021a) which in turn provides direct access to a large number of concept lists that have been proposed in the past, it is not difficult to compare to which degree concepts which have been assigned to lists of supposedly stable items behave differently with respect to the automatically inferred borrowings in this sample.

Two supposedly stable concept lists with a high resistance to borrowing are Swadesh's list of 100 items ( Swadesh, 1955) and the so-called Leipzig-Jakarta list derived from the World Loanword Database which lists manually identified borrowings for a sample of 41 genetically diverse language varieties ( Haspelmath & Tadmor, 2009). In Table 4, we have calculated the average number of non-borrowed items (words which occur uniquely in one language variety and words which are shared within one language family alone) for the traditional Swadesh list of 100 items, the Leipzig-Jakarta list of 100 items ( Tadmor, 2009), their respective counterparts (the subset of items which do not occur in the 100-item Swadesh list and the Leipzig-Jakarta list), as well as the base list of 250 concepts. As we can see from the table, there is a considerable difference in terms of supposed stability (or resistance to borrowing) when comparing the supposedly stable, borrowing-resistant sublists with their respective counterparts. While the amount of supposedly borrowed words in this sample may seem to be remarkably high, exceeding 15% for both the sublists and the list of all concepts, it should be kept in mind that our approach does not control for directions and inheritance. Since the source and target words of borrowings are not distinguished, the numbers do not indicate the amount of borrowed words, but the amounts of *xenologs*, that is, sets of etymologically related words which have experienced lateral transfer events in their past (see List, 2016).

**Table 4.** Proportion of potential borrowings in the data and various sublists.

Concept list	Proportion of non- borrowed items	Number of items
Swadesh (1955)	0.80	78
No Swadesh	0.70	172
Leipzig-Jakarta	0.78	61
No Leipzig-Jakarta	0.72	189
All items	0.73	250

In order to test whether the observed differences between the proportions of xenologs are significant, or whether they could have alternatively arisen by chance, we ran 10000 trials in which the concept list was split into two parts, reflecting the proportion of the Swadesh list (with 78 items vs. 172 items) and the Leipzig-Jakarta list (61 items vs. 189 items). In all trials, we tested whether one could observe the same or a higher difference between the amount of non-borrowed items and potential xenologous words. The results suggest that it is not very likely to obtain the differences for the Swadesh list by chance. We obtained similar differences in only 3% of all cases. For the Leipzig-Jakarta list, however, the results were slightly different, and we obtained similar results in 7.1% of all trials. While this number is still low, it would not pass a classical significance test.

**Figure 5.**
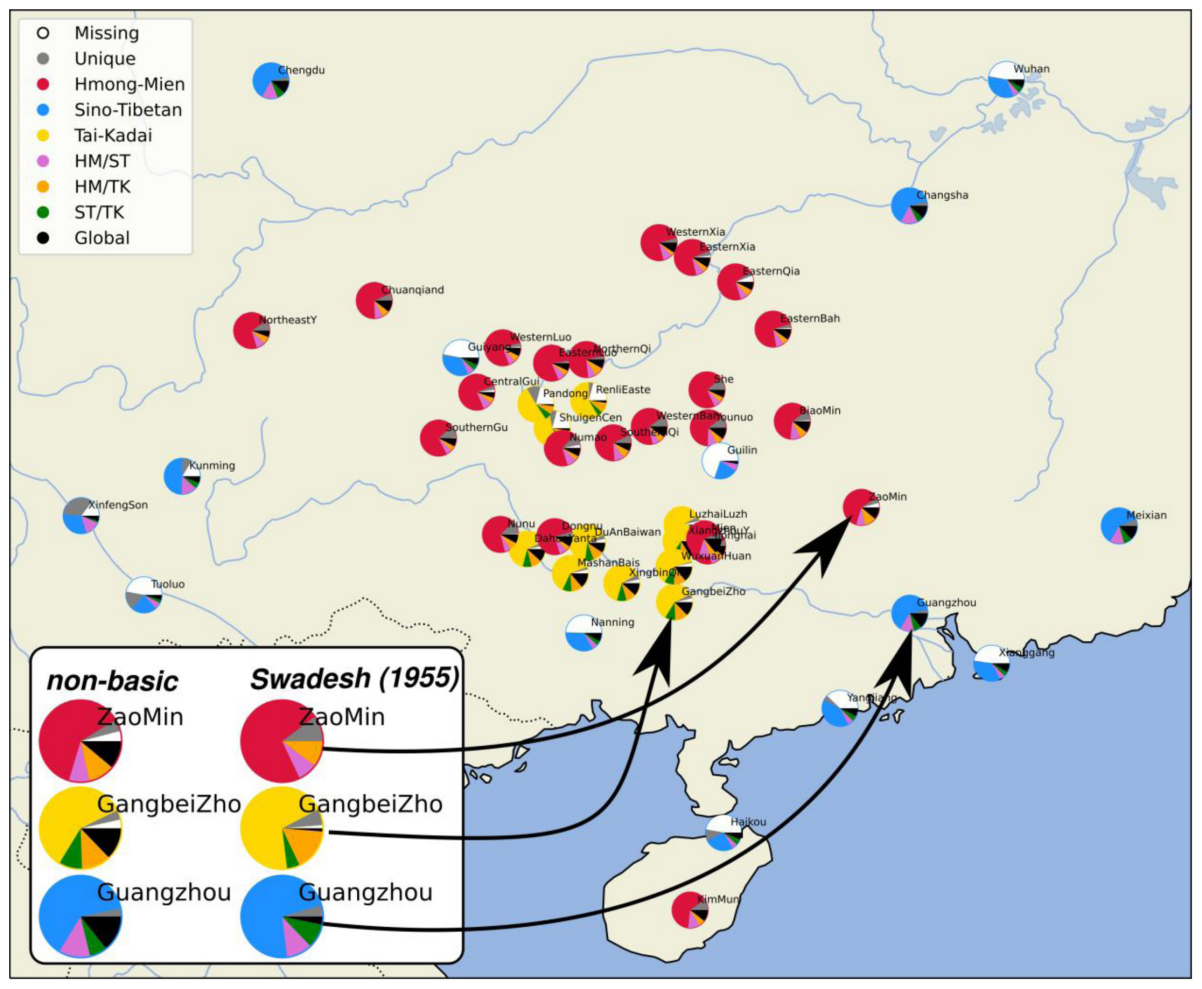
Admixture plots of shared lexemes between the major language families.

That there are — at times even striking — differences between supposedly stable concepts and concepts more prone to borrowing can also be directly seen when visualizing the characteristics of individual words in each language with the help of geographic plots inspired by "admixture plots" in genetics ( Pritchard *et al.*, 2000). In this visual representation, we inspect the words in each language in separation and distinguish (1) *missing data* (no word form for a given concept available), (2) *singletons* (words occur only in this specific variety), and (3) *language-family-internal words* (words that are cognate with words from related languages), from (4) *words shared among two language families* (e.g., Sino-Tibetan vs. Tai-Kadai), and (5) *words shared among all three language families* in our sample. Such a plot is shown in Figure 5 for all 250 concepts in the sample. On the bottom left of the figure, three varieties (Zao Min, Gangbei Zho, and Guangzhou) have been additionally contrasted with respect to the distribution that they would show for the non-basic items of the 250 concept list and the basic items from Swadesh's list from 1955. As can be seen, all varieties show a much-increased amount of etymologically non-relatable singletons and language-family-internal cognates.

### Examples

While numbers and plots can to some degree help us to assess how well a certain method works, it is always important to inspect individual case studies as well in order to explore where the specific weaknesses of a method lie and how this could be overcome in future work. While the EDICTOR interface, introduced earlier, already greatly facilitates the manual inspection and correction of automatically generated cognate judgments (including judgments on potential borrowings), specifically for the detection of borrowings it can be useful to inspect the data in geographic space. For this reason, we created a small routine which plots the inferred sets of words shared across more than one language family on a map and contrasts them with those words which were not assigned to the same cluster.

**Figure 6.**
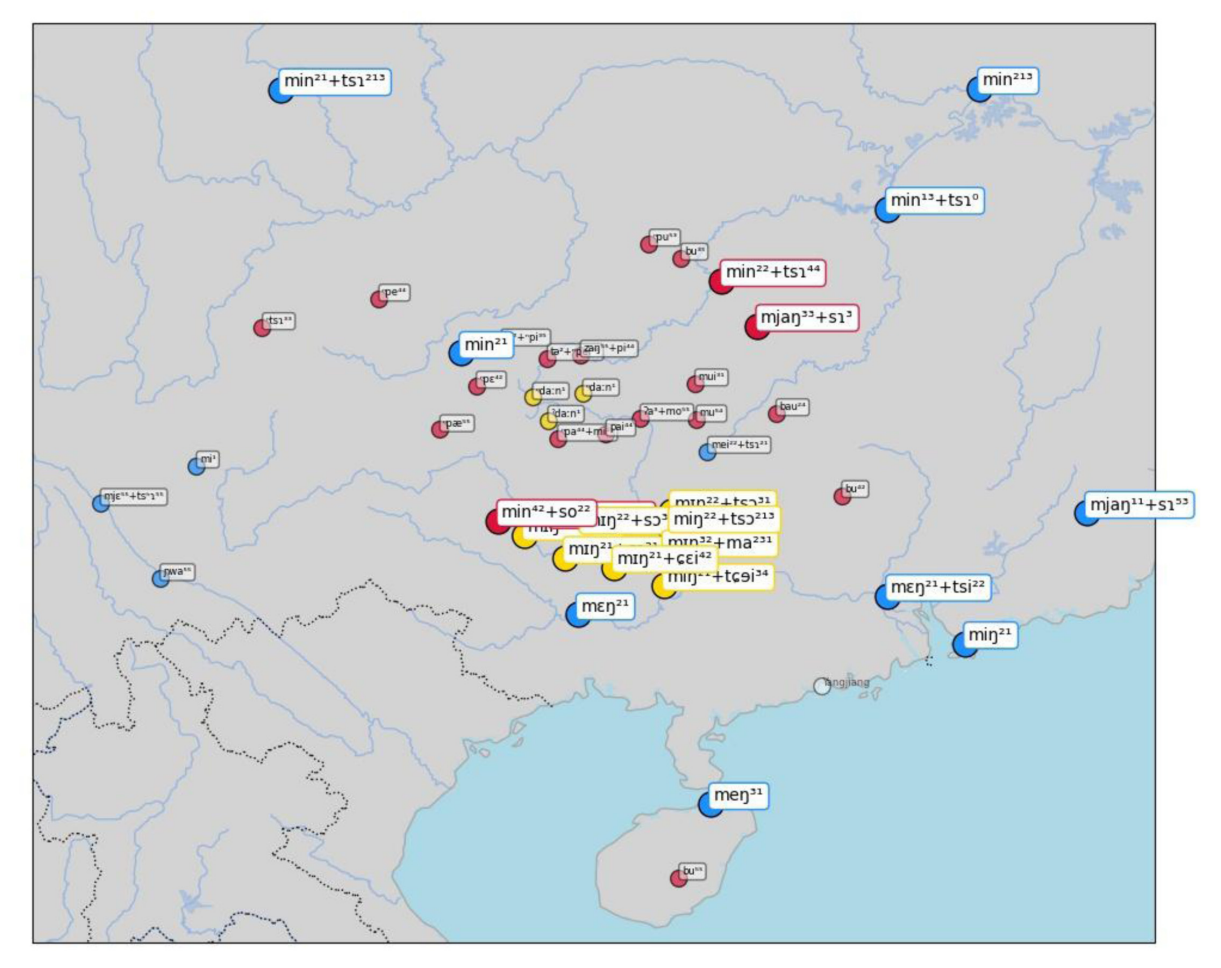
Automatically inferred cluster of potential borrowings for "name".

A first example of this visualization can be seen in Figure 6, showing inferred sets for the concept "name", which are — as we know well — all borrowed from Chinese *míngzì* 名字 (Middle Chinese *mjieng dziH*). While most of the cases inferred by the algorithm are striking (all Tai-Kadai languages have almost literal copies of the Mandarin form), we also find a couple of surprising cases of false negatives in this sample. Thus, the word form [m ei ²² + ts ɿ] in the Chinese dialect of Guilin (15 in the center of the map) clearly belongs to the cluster, as does the form [m j ɛ ⁵⁵ + ts ʰ ɿ] in Xinfeng Sonaga (a Loloish variety of Sino-Tibetan), or the form [m i ¹] in Tuluo Bai (a Sino-Tibetan variety whose deeper affiliation remains unclear so far). Since there are two forms in which the Chinese word for "name" can be borrowed, as simplex form *míng* 名, meaning "name", which points to more archaic borrowing events, and in the modern Mandarin form *míngzì* 名字, lit. meaning "name sign", and since the major cluster consists of the bisyllabic form, the algorithm for sequence alignment has problems of identifying short words which lack the final nasal as belonging to the cluster.

**Figure 7.**
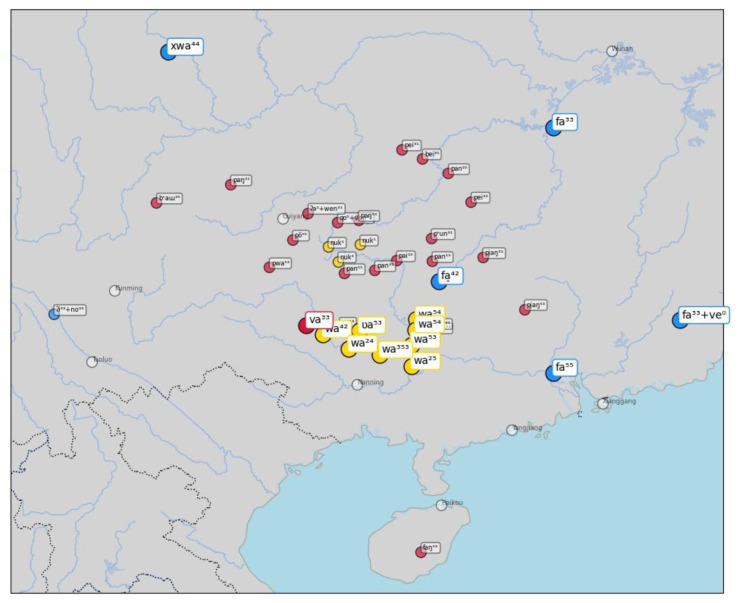
Automatically inferred cluster of potential borrowings for "flower".

As a second example, consider words for "flower" in Figure 7. Here, the algorithm correctly identifies the similarity between forms like [w a ²⁴] in the Zhuang varieties of Tai-Kadai, which also occurs in the Hmong-Mien variety Nunu as [v a ³³] and is a rather obvious borrowing from Chinese *huā* 花 (Middle Chinese *xwae*), which shows the sound change [xw] > [f] in many Southern varieties of Chinese.

**Figure 8.**
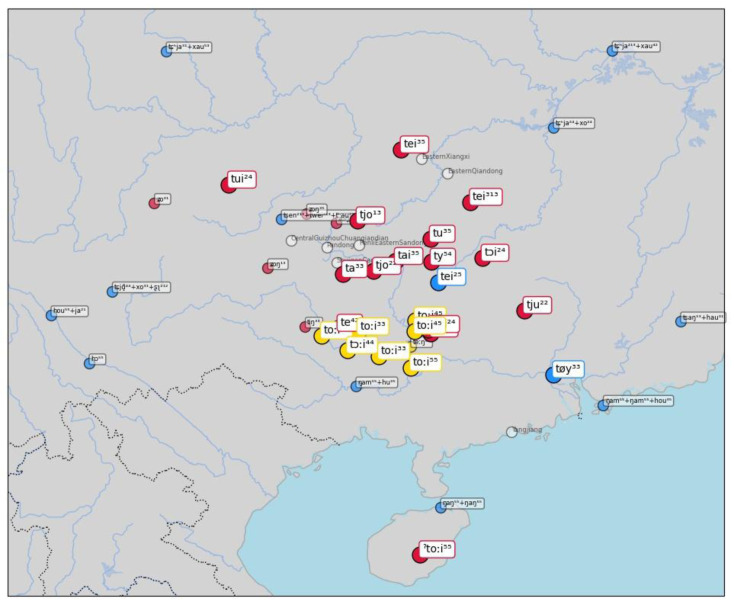
Automatically inferred cluster of potential borrowings for "correct (right)".

As a last example, consider similar word forms for "correct (right)" in Figure 8. Here the algorithm clusters word forms such as [t ɔi] in BiaoMin (from the Mienic branch of Hmong-Mien) and [t oːi ⁴⁴] in DahuaYantan (from the Zhuang branch of Tai-Kadai). The source word, however, is again from Chinese, where *duì* 對 (Middle Chinese twoijH) is still the basic way to express "correct (right)" in Mandarin Chinese and many other Chinese dialect varieties. The majority of the sources in the sample selected here provide different words in the Chinese dialects, and we find expressions such as [ts ə n ¹³ + x au ⁵³] (Mandarin Chinese *zhēnhǎo* 真好 "totally right") in Chengdu. The fact that we find different word forms in the data does not mean, however, that there have been recent events of lexical replacements in many Chinese dialects. It seems instead that this variation is due to the elicitation process. Since the dialect data for Chinese comes from a variety of sources, it is either possible that the mapping of the concepts to the Concepticon project is not entirely correct, or it may be due to the fact that the elicitation process forced the use of longer or more specific expressions, different from the extremely common *duì*.

It would go beyond the scope of this study to discuss all individual findings made by the algorithm in detail. All plots, however, are shared along with the supplementary material accompanying this study, so that interested readers can dive into the individual results and criticize and improve them. What is important to note, however, is that the inspection has shown that there are definite points where the method proposed here could be further improved. The major problem of the scoring procedure used is that it is very sensitive to phonotactic representations. As a result, two-word forms which sound rather similar but are represented phonotactically quite different, can be easily judged to be unrelated for the scoring procedure, while human linguists immediately spot the overall similarity. As an example, consider the form [t w əi ⁵¹] which is one possible way to represent the word *duì* "correct" in Mandarin Chinese and the form [t oːi ⁵⁵] in Gangbei Zhongli (Zhuang branch of Tai-Kadai). Phonotactically (for the scoring function), the first form consists of two consonants and one vowel, while the second form only consists of one consonant and one vowel. While humans judging the similarity between both forms would probably ignore the medial [w] as being irrelevant and put more emphasis on the striking match of the diphthongs, the distance scoring employed by the method here is not yet capable of providing such a fine-grained weighting. Alternative approaches for sequence alignments would be needed to detect the particular similarity between the two words, but this is not necessarily trivial, since it would require us to loosen our strict distinction between vowels and consonants, which is crucial in most alignment approaches (including those based on the computation of edit distances, see e.g., Prokić *et al.*, 2009). 

## Conclusion

Although there has been a lot of research in the field of computational historical linguistics of late, no major improvements in the field of automated borrowing detection have been made so far. The method proposed in this study is very simple and can only detect potential borrowings between languages from different language families. However, as we have tried to show, the method can still be quite useful, both for the automated investigation of contact phenomena in large lexical datasets, and for the more detailed development of computer-assisted case studies, where the method can be used to preprocess the data in order to make the manual annotation more efficient. We therefore consider the new method as a first step towards a more intensive treatment of language contact phenomena in the field of computational historical linguistics.

More detailed case studies will be needed to fully test the potential of our approach. One interesting test case we can think of, could consist of a more detailed study of South-East Asian languages including languages beyond our sample from Southern China, accounting for the observation that specifically Tai-Kadai languages outside of China differ greatly from those inside China, due to the different degrees of Sinitic influence ( Szeto & Yurayong, fortcoming). Additional case studies can be carried out with any linguistic area where a sufficient amount of languages from different language families are spoken and lexical data in phonetic transcription for a sufficiently large number of concepts are available.

## Data availability

Zenodo: CLDF dataset accompanying List and Forkel's "Borrowing Detection in Multilingual Wordlists" from 2021. https://doi.org/10.5281/zenodo.5037101 ( List & Forkel, 2021b)

Data are available under the terms of the Creative Commons Attribution 4.0 International license (CC-BY 4.0).

Concrete instructions on reproducing the study can be found at https://github.com/lexibank/seabor/blob/v1.0/workflow.md.

## Software availability

**Source code available from:** https://github.com/lexibank/seabor

**Archived source code at time of publication:** https://doi.org/10.5281/zenodo.5037100 ( List & Forkel, 2021b)

**License:** Creative Commons Attribution 4.0 International
