# Supplementary material for: Automated identification of borrowings in multilingual wordlists
Source: Open Res Eur. 2022 Mar 23;1:79. Originally published 2021 Jul 15. [Version 3] doi: 10.12688/openreseurope.13843.3 (PMC10445856; doi:10.12688/openreseurope.13843.3)
Supplement: Peer review history

# Peer review: Reviewer response for version 2

**DOI:** 10.21956/openreseurope.15171.r27472

**Published:** 2021-09-02

**Authors:** John Nerbonne

**Affiliations:** 1 Computational Linguistics and chair of Humanities Computing, University of Groningen, Groningen, The Netherlands

I find this much clearer, and I approve of its indexing without reservation.

 It's a strong paper about an important topic.

 I noted a couple of typos, problems in font embedding, and slips in diction, however.

 p.5 "requires to search" => requires one to search"

 p.8 "borrowing sare" => borrowings are

 p.11 "considerably small" => rather small

 p.18, phon. transcription not rendered in .pdf

 Finally, I would have appreciated some speculation on WHERE the improvement comes from, but I wouldn't suggest holding anything up for that reason.

If any, are all the source data and materials underlying the results available?

Yes

Is the work original in terms of material and argument?

Partly

Is the work clearly and cogently presented?

Partly

Is the argument persuasive and supported by evidence?

Yes

Does the research article contribute to the cultural, historical, social understanding of the field?

Yes

Does it sufficiently engage with relevant methodologies and secondary literature on the topic?

Yes

Reviewer Expertise:

NA

I confirm that I have read this submission and believe that I have an appropriate level of expertise to confirm that it is of an acceptable scientific standard.

# Response

**Published:** 2021-09-02

**Authors:** Johann-Mattis List

**Affiliations:** University of Passau, Germany

We are very grateful for the assessment by the reviewer and will try to submit a third version of our article soon which makes up for all points raised.

# Peer review: Reviewer response for version 1

**DOI:** 10.21956/openreseurope.14926.r27264

**Published:** 2021-08-12

**Authors:** Kenichi W. Okamoto

**Affiliations:** 1 Department of Biology, University of St. Thomas, St. Paul, MN, USA

This manuscript introduces and illustrates a new technique for identifying borrowing events found both within and across multiple language families. While computational approaches are a well-suited and increasingly popular tool for exploring language change when the underlying lexical data are likely orthologous, linguistic borrowing presents a major and very real complication. For many languages the phenomenon is often poorly attested in the historical record. It is sufficiently common to potentially bias several standard tools from systematic biology for assessing phylogenies. These challenges are exacerbated by the difficulty of manually assessing the evolutionary fate of proto-words across several languages to distinguish xenologs from orthologs; doing so requires expertise in the idiosyncratic evolution of several, often unrelated lineages.

 Here, the authors aim to make some headway viz. the difficulty of identifying borrowing events when multiple language families are involved. Briefly, the authors propose a two-step procedure, first identifying cognates within language families and then seeking to identify cognates across families. The authors then provide a case study and examine patterns of lexical borrowing within a well-studied Sprachbund of three language families in southeast China. The soundness of the proposed approach is assessed through well-established text clustering methods. They then introduce several techniques by which comparative linguists might begin to quantitatively characterize how language contact develops. 

 General comments:

 I find this last aspect of the study particularly useful, as it moves the study of Areal linguistics in a productive direction. For instance, their use of admixture plots not only allows linguists to visualize patterns of borrowings spatially; it isn't hard to envision extensions that can map similar measures onto features of physical geography (for instance) in a way that can yield insights previously hard to explore statistically. These approaches have proven a boon to our understanding of how gene flow interacts with ecological processes in biogeography. Whether such extensions in linguistics will materialize remains to be seen, but on balance my sense is the applications described here lay a plausible point of departure for quantitatively and I think usefully comparing language contact dynamics across Sprachbunds.

 Specific comments:

 Background: 

 It wasn't clear what was meant by "shortcut status" here.

 Materials:

 Second paragraph, "Having shown..." the authors note "the dialect data do not provide strict information on actual words" - it was admittedly unclear to me why this was so. How plausible is it that the phonetic properties of the morphemes/characters change sufficiently to drown out the cross-dialectical signal in the context of different words? Admittedly, I'm not familiar enough with subtle morpheme distinctions in Sino-Tibetan to have a good feel about this, but maybe some clarification/references on this point would help. 

 Methods:

 Second paragraph: "For our specific use case..." versus "Since one would expect, ...". I wondered if this contrast means that the use of full cognates should be the more parsimonious starting point when applying the approach to other contexts, or if the search for partial cognates makes more sense? Presumably there is some degree of compounding in the morphology of many languages that complicate (esp. automated) attempts at identifying borrowing patterns. Could the authors comment on the advisability of using partial rather than full cognates during the first step beyond their case study?

 Third paragraph: "If the average distance ..." are there any rough rules of thumb on reasonable thresholds on which the authors can comment? Particularly perhaps based on their manual comparisons later.

 "Specific Results":

 Although I didn't have immediate access to List 2014, presumably the "True chain" (sensu Bagga and Baldwin 1998) consists of the manually annotated cognates/borrowings (analogously with the F-statistic's True/False positives)? I suspect a quick clarification would help. Also, unlike the rest of the manuscript, this segment alone appears written in the first person.

 Table 2: Since it won't be at all computationally costly, it might be interesting to see what a bootstrapped or other resampling analysis of the evaluation metrics would show viz. characterizing the variability around the test statistics.

 Third paragraph: "In order to test...": I may have missed something in the description of the resampling test here, but a caveat is that this analysis involves multiple comparisons across two lists (Swadesh/Leipzig-Jakarta), so the significance test threshold mentioned at the end likely can be adjusted. Granted, the proportion is still quite low for both lists even when doing so, which I suspect is still linguistically meaningful.

 Finally, as I am not particularly familiar with the three language families analyzed, I cannot comment on how reasonable the present study's findings will strike a specialist in the language families explored. I have, however, studied the underlying workflow code, and the authors' implementations appear technically sound as far as I can tell.

If any, are all the source data and materials underlying the results available?

Yes

Is the work original in terms of material and argument?

Yes

Is the work clearly and cogently presented?

Yes

Is the argument persuasive and supported by evidence?

Yes

Does the research article contribute to the cultural, historical, social understanding of the field?

Yes

Does it sufficiently engage with relevant methodologies and secondary literature on the topic?

Yes

Reviewer Expertise:

Evolutionary biology, computational biology

I confirm that I have read this submission and believe that I have an appropriate level of expertise to confirm that it is of an acceptable scientific standard.

## References

[1] : Entity-based cross-document coreferencing using the Vector Space Model. *ACL '98/COLING '98* .1998;1: 10.3115/980845.980859 79-85 10.3115/980845.980859

# Response

**Published:** 2021-08-12

**Authors:** Johann-Mattis List

**Affiliations:** University of Passau, Germany

We thank the reviewer for taking the time for this very thorough review. What is specifically nice is that the reviewer -- due to the different background -- expands the perspectives on our study even more, providing some new suggestions that will help us to improve the study further.

# Response

**Published:** 2021-08-19

**Authors:** Johann-Mattis List

**Affiliations:** University of Passau, Germany

Dear Kenichi W. Okamoto, The revised version of the manuscript which we just submitted hopefully addresses the points you raised in your review. We list out individual responses to the points in bullet point form below.

- *It wasn't clear what was meant by "shortcut status" here.*
  We have clarified this now.
- *Table 2: Since it won't be at all computationally costly, it might be interesting to see what a bootstrapped or other resampling analysis of the evaluation metrics would show viz. characterizing the variability around the test statistics.*
  We have expanded the evaluation measures in the general analysis now drastically, and this has helped us to clarify for ourselves what the particular benefits of the method are. Especially the introduction of the baselines proved important. For bootstrapping or similar evaluations, we were not sure how to proceed in concrete, but hope that our new tests are satisfying enough.
- *Although I didn't have immediate access to List 2014, presumably the "True chain" (sensu Bagga and Baldwin 1998) consists of the manually annotated cognates/borrowings (analogously with the F-statistic's True/False positives)? I suspect a quick clarification would help. Also, unlike the rest of the manuscript, this segment alone appears written in the first person.*
  We have extended this part, not in detail, but by showing what the measures do. We refer to a page range in the book by List (2014). Since the book is open access, readers interested in understanding these evaluation measures can now find all the necessary information needed.
- *Third paragraph: "In order to test...": I may have missed something in the description of the resampling test here, but a caveat is that this analysis involves multiple comparisons across two lists (Swadesh/Leipzig-Jakarta), so the significance test threshold mentioned at the end likely can be adjusted. Granted, the proportion is still quite low for both lists even when doing so, which I suspect is still linguistically meaningful.*
  We discussed if resampling would apply, but have to admit that it is not entirely clear to us, if this applies in our examples, where we merely compare significance scores among two different datasets and do not seek to find overall significant results. We try to avoid to fix a significance value (which would, if we understand Bonferroni correction well, be 0.025 due to the fact that we have two tests), so we just left out this part, where we mention the 5%, which are anyway disputed among scholars. We hope that the major message here (that Swadesh (1955) splits the dataset with the automated borrowings more meaningful than the Leipzig-Jakarta list) is still correct in this form, even if we avoid to open a discussion about significance values.

# Peer review: Reviewer response for version 1

**DOI:** 10.21956/openreseurope.14926.r27266

**Published:** 2021-08-06

**Authors:** George S. Starostin

**Affiliations:** 1 Institute of Oriental and Classical Studies, Russian State University for the Humanities, Moscow, Russian Federation

The paper is a serious step forward in the development of new techniques to automatically identify potential borrowings between groups of (assumedly) unrelated languages. The new method follows the authors' earlier research on similar algorithms for eliciting actual cognates between (assumedly) related languages and is largely operating along the same lines. The selected data set upon which the algorithm has been tested is valid, though in the future it would be essential to expand the test base by data from other areas of the world to confirm its universal applicability.

 I have detected one serious (though local) mistake in the main bulk of the paper: the MC reconstruction for the word 'face' is given as *mjuwk, when in reality that is the reconstruction for 'eye' (目 mu); the proper MC reconstruction for 'face' is *mjienH, which is, of course, the form that is phonetically compatible with both the modern Sinitic reflexes and the borrowed HM forms. This should by all means be corrected, and perhaps the other MC forms in the data should be double-checked as well.

 All in all, the work definitely merits indexing.

If any, are all the source data and materials underlying the results available?

Yes

Is the work original in terms of material and argument?

Yes

Is the work clearly and cogently presented?

Yes

Is the argument persuasive and supported by evidence?

Yes

Does the research article contribute to the cultural, historical, social understanding of the field?

Yes

Does it sufficiently engage with relevant methodologies and secondary literature on the topic?

Yes

Reviewer Expertise:

Comparative-historical linguistics, Chinese historical linguistics

I confirm that I have read this submission and believe that I have an appropriate level of expertise to confirm that it is of an acceptable scientific standard.

# Response

**Published:** 2021-08-06

**Authors:** Johann-Mattis List

**Affiliations:** University of Passau, Germany

We thank the reviewer a lot for the review, and specifically for catching the indeed serious if not even embarrassing error regarding the pronunciation of Middle Chinese "face", which we will of course correct. This shows how important it is when dealing with multi-disciplinary topics that reviewer experts from different fields check a study for particular problems.

# Response

**Published:** 2021-08-19

**Authors:** Johann-Mattis List

**Affiliations:** University of Passau, Germany

Dear George S. Starostin, Our revised version now lists the correct form for "face". We are very thankful that you caught this mistake. When looking up the character reading in Baxter (1992), we must have strangely swapped the characters, but this has been corrected now.

# Peer review: Reviewer response for version 1

**DOI:** 10.21956/openreseurope.14926.r27263

**Published:** 2021-07-30

**Authors:** John Nerbonne

**Affiliations:** 1 Computational Linguistics and chair of Humanities Computing, University of Groningen, Groningen, The Netherlands

**Summary**

 The paper proposes a two-step process for identifying borrowings. First, one applies earlier work by the authors to obtain language-family-internal cognate sets, and second, it applies a sound-class alignment algorithm (SCA) due to the first author. The paper evaluates using a substantial data set and reports F1-scores between 0.86 (for detecting borrowing) and 0.88 (for detecting cognacy).

**Comments**

 The paper reports on work advancing the detection of loan words, which is an important issue, and it seems to be successful, so I recommend that it be indexed, but I urge the authors to consider the remarks below. The use of the admixture plots in Fig. 3 is a clear advance, and Table 3, noting the relatively low proportion of non-borrowed words in the Swadesh list, will be extremely interesting to all historical linguists!

 I had the advantage of reading Pui Yiu Szeto’s review, which is fortunate since I would not presume to comment on the quality of the work with respect to Sinology. But where Szeto praises the article’s accessibility, I would urge the authors to make the work even more easily accessible to specialists and non-specialists.

 Most of my complaints concern clarity, and given the (laudable!) availability of both data and software, might not bother readers who consult all the available material, but there’s something to be said for providing information in a relatively self-contained paper.

 I assume that input data is the usual table of concepts (rows) and varieties/doculects (columns) (or a 90° rotation of that), but it would help to be explicit. It is unclear to me whether input language data is annotated with respect to its putative language family (going beyond the usual input data), as Fig. 2 suggests. Identifying families is often part of the task, of course.

 The notion ‘partial cognate’ is crucial, but neither defined nor illustrated. I’d suggest doing both, even if this repeats material from List et al. 2016). In the same vein, it would help to illustrate the two steps on a small data set.  These two points should be addressed.

 The clarity of the paper might also improve if the authors would explain (and illustrate) step one thoroughly and then turn to step two (in the “Methods” section). As it now stands, the first para. explains both steps, while the second returns to the first step. Page 4 reports that partial cognates were converted to full cognates (in the first step) using a variety of the LexStat algorithm as modified recently in (Wu & List submitted) that “assigns partial cognates to full cognate sets which have at least one cognate set in common”. The idea is to recover cognate morphemes, even when these only occur within words, e.g. Dutch *moestuin* /’mu:s.tœyn/ ‘vegetable garden’ is compared with German *Gemüse* /gə.’my:.zə/ ‘vegetable’, which share the morpheme {mUS} (an example I could think of quickly, but if the authors wish to illustrate the concept, as I suggest, then using Chinese data would probably be preferable. While I have a vague idea what the technique must be doing (a sort of Longest Common Subsequence algorithm?), it would help to at least illustrate it concretely, especially since the cited source still awaits publication, and since its focus seem to be on annotation, not (partial) cognate detection. ^1 ^I wouldn’t mind reading a dataflow diagram with example data. 

 The second step uses List’s SCA algorithm, as noted in the summary, which tests pronunciation similarity with respect to the question of common genealogy, asking whether two words are likely to have descended from the same source – either via ancestry or borrowing. All word pairs within putative cognate sets are tested and the sets are identified as involving borrowing the average distance falls below a user-defined threshold. This is novel, but I would urge the authors to speculate on whether this is the source of the improvement they demonstrate. Comparing all pairs of words based on SCA distance would follow an obvious step from earlier work (Zhang *et al* 2021), but the advantage of the approach in this paper might be in comparing cognate *sets* with respect to similarity rather than just individual words from the two different languages. It would be good to hear from the authors, especially if they are of a different opinion. see below.

 It is also unclear how the “user-defined threshold” (p.4) is determined. These issues should be clarified, since they are relevant with respect to how independently the procedures work (independent of annotation, and independent of user-supplied parameters), and how generally applicable they can be.

 The authors are candid about their techniques not indicating the direction of borrowing, but I assume that it will be difficult to detect cases where two languages borrow from a third, such as the word ‘sputnik’ in many languages. The issue might be made explicit.

 The sensitivity to phonotactics (noted on p.10) is interesting, since the example is the sort that edit distance approaches have little difficulty with. Perhaps the authors would care to comment further.

 The authors might wish to refer to neighbor net and splits-tree as potentially useful ways of analyzing the historical data once borrowings have been determined.

 Form: The paper is very readable, but there are a couple of slips.

- withour => without (caption, Tbl.1)
- concepts was selected => were (p.4, l.1)
- amount of words, etc. => number of words (passim); more conservative readers use ‘amount’ only with mass nouns. So this may be a reactionary recommendation.
- Zhang et al. now has page numbers: 269-288 and has been announced for appearance in Oct. (References)

Conclusion: The paper should be indexed after the requests for clarifying remarks have been addressed.  These concern (i) what partial cognates are; (ii) how the revised LexStat algorithm works, including an example application on illustrative data; and (iii) whether comparing average distances between potential cognate sets is the source of the improvement in detection.

1. I looked at Wu & List (submitted) without reading it closely, but it didn’t clarify this point for me. Perhaps the technique might be identified more specifically via page numbers or name.

If any, are all the source data and materials underlying the results available?

Yes

Is the work original in terms of material and argument?

Partly

Is the work clearly and cogently presented?

Partly

Is the argument persuasive and supported by evidence?

Yes

Does the research article contribute to the cultural, historical, social understanding of the field?

Yes

Does it sufficiently engage with relevant methodologies and secondary literature on the topic?

Yes

Reviewer Expertise:

Computational linguistics, comparative linguistics

I confirm that I have read this submission and believe that I have an appropriate level of expertise to confirm that it is of an acceptable scientific standard, however I have significant reservations, as outlined above.

# Response

**Published:** 2021-08-04

**Authors:** Johann-Mattis List

**Affiliations:** University of Passau, Germany

We thank the reviewer a lot for taking the time for this very thoughtful and very detailed report. We will try to address these concerns soon after we have received one more review report which may point to additional problems in our approach which have not been covered so far.

# Response

**Published:** 2021-08-19

**Authors:** Johann-Mattis List

**Affiliations:** University of Passau, Germany

Dear John Nerbonne, we have now revised the manuscript and submitted an updated version, in which we tried to account for all changes you suggested. Below, we list our modifications in the form of bullet points.

- *I assume that input data is the usual table of concepts (rows) and varieties/doculects (columns) (or a 90° rotation of that), but it would help to be explicit. It is unclear to me whether input language data is annotated with respect to its putative language family (going beyond the usual input data), as Fig. 2 suggests. Identifying families is often part of the task, of course.*
  We have added a workflow figure in which we hope that the algorithms are clarified sufficiently now.
- *The notion ‘partial cognate’ is crucial, but neither defined nor illustrated. I’d suggest doing both, even if this repeats material from List et al. 2016). In the same vein, it would help to illustrate the two steps on a small data set.  These two points should be addressed.*
  We hope our Figure 2 will address this question now, we also add clarification in Figure 3, and we refer to additional literature. We emphasize that while we could provide many more examples in this study, we consider that it would lead too far away if we had to comment on all ongoing debates on partial cognates. For this reason, we now provide more examples but also refer to the literature which is in our impression crucial.
- This is novel, but I would urge the authors to speculate on whether this is the source of the improvement they demonstrate. Comparing all pairs of words based on SCA distance would follow an obvious step from earlier work (Zhang et al 2021), but the advantage of the approach in this paper might be in comparing cognate *sets* with respect to similarity rather than just individual words from the two different languages. It would be good to hear from the authors, especially if they are of a different opinion. see below.
  We explicitly comment on this now and contrast our approach with an earlier approach where no direct evaluation was, however, provided.
- *It is also unclear how the “user-defined threshold” (p.4) is determined. These issues should be clarified, since they are relevant with respect to how independently the procedures work (independent of annotation, and independent of user-supplied parameters), and how generally applicable they can be.*
  We discuss the difficulties of finding thresholds in our methods section now.
- *The authors are candid about their techniques not indicating the direction of borrowing, but I assume that it will be difficult to detect cases where two languages borrow from a third, such as the word ‘sputnik’ in many languages. The issue might be made explicit.*
  The method can detect all those cases where cognates spread several language families. If sputnik only occurred in Hmong-Mien languages, but not in Tai-Kadai and Chinese, we would not be able to detect the word, but if it occurs across two or more language families, we should be able to find it.
- *The sensitivity to phonotactics (noted on p.10) is interesting, since the example is the sort that edit distance approaches have little difficulty with. Perhaps the authors would care to comment further.*
  We address this now explicitly in the results section, emphasizing that it is the matching of vowels and consonants, which is problematic here. We call this “phonotactics”, which may be missleading, but we hope that our additional statement clarifies the problem.
- *withour => without (caption, Tbl.1)*
  We corrected this, thanks.
- *concepts was selected => were (p.4, l.1)*
  We corrected this, thanks.
- *amount of words, etc. => number of words (passim); more conservative readers use ‘amount’ only with mass nouns. So this may be a reactionary recommendation*.
  We corrected this, thanks.
- *Zhang et al. now has page numbers: 269-288 and has been announced for appearance in Oct. (References)*
  We corrected this, thanks.
- *These concern (i) what partial cognates are;*
  We hope, our response above and the revised manuscript make this clearer now.
- *(ii) how the revised LexStat algorithm works, including an example application on illustrative data; and*
  We also hope to have clarified this point.
- *(iii) whether comparing average distances between potential cognate sets is the source of the improvement in detection.*
  We hope our additional statistics on thresholds provide the necessary insights here.

# Peer review: Reviewer response for version 1

**DOI:** 10.21956/openreseurope.14926.r27265

**Published:** 2021-07-21

**Authors:** Pui Yiu Szeto

**Affiliations:** 1 Department of Linguistics, The University of Hong Kong, Hong Kong, China

This article introduces a new automated method for detecting lexical borrowing in contact scenarios involving multiple languages of different genetic affiliations. Although there have been a number of studies on automated detection of lexical borrowing over the past decade, this study represents a major breakthrough since this is the first attempt to apply this approach to more than two languages at the same time.

 Overall speaking, this is a very informative and well-organized article. The authors manage to provide a clear and concise introduction to the background information of the subject matter. The methodology part of studies concerning computational methods often looks rather daunting to non-specialists, but this article presents everything in a reader-friendly manner, while providing the relatively technical details in the supplementary materials for those who are interested in replicating the experiments or testing the method on a different set of data. Granted, like other automated methods in computational historical linguistics, the results generated by this new method must be checked by historical linguists with specialist knowledge of the languages concerned; nonetheless it appears to be a very handy tool for the study of lexical borrowing in contact scenarios.

 There are some minor issues which the authors may want to take into consideration when they carry out further studies along this direction.

1. Reading the abstract, one will normally expect that languages spoken in Southeast Asian countries like Vietnam, Cambodia, Thailand, and Laos are considered in this study. However, all the languages involved are actually spoken in Southern China. Apart from being potentially misleading, a problem of exclusively focusing on languages in Southern China is that there may not be sufficient language-family-internal variation in terms of borrowing patterns to yield interesting results, as shown in the admixture plots of Tai-Kadai and Hmong-Mien languages in Figure 3. Given the considerable structural differences between Tai-Kadai languages spoken inside and outside China due to varying degrees of Sinitic influence (Szeto and Yurayong [forthcoming]), it will be interesting to incorporate at least a couple of Tai-Kadai languages spoken outside China to see whether they display a remarkably different pattern of lexical borrowing. If this is the case, it may suggest that the method can even help access the intensity of language contact.
2. The authors rightly point out that the Chinese dialect data in this study is less than ideal. First, there are many missing concepts in some of the dialects. More importantly, the data comes from various sources, subject to non-uniform (and sometimes questionable) elicitation processes. A case in point is the word form for 'correct'. In Yue dialects , the most common form is *ŋam* 啱, which is possibly a loanword of Tai-Kadai origin (Li 1990). I suggest using the *Great Dictionary of Modern Chinese Dialects* (Li 2002) as the sole source of Chinese dialect data. Li (2002) is a compendium of dictionaries for 42 Chinese dialects following a uniform format. This can surely improve the accuracy and coverage rate of the Chinese dialect data.

If any, are all the source data and materials underlying the results available?

Yes

Is the work original in terms of material and argument?

Yes

Is the work clearly and cogently presented?

Yes

Is the argument persuasive and supported by evidence?

Yes

Does the research article contribute to the cultural, historical, social understanding of the field?

Yes

Does it sufficiently engage with relevant methodologies and secondary literature on the topic?

Yes

Reviewer Expertise:

Language contact, linguistic typology, language change, East and Southeast Asian languages

I confirm that I have read this submission and believe that I have an appropriate level of expertise to confirm that it is of an acceptable scientific standard.

## References

[1] : Establishing a Sprachbund in the Western Lingnan region: Conceptual and methodological issues source.(forthcoming);

[2] : 粤语中的壮侗语族语言底层初析 [A preliminary analysis of Tai-Kadai substrate words in Yue]. *中央民族学院学报 [Journal of the Central Institute for Nationalities]* .1990;71-76.

[3] : 現代漢語方言大詞典 [The great dictionary of Modern Chinese dialects]. *Jiangsu Education Publishing House* .2002;

# Response

**Published:** 2021-07-27

**Authors:** Johann-Mattis List

**Affiliations:** University of Passau, Germany

We thank the reviewer very much for these very valuable and also inspiring comments. We will respond to them in more detail later, when more reviews have been received and also try to account for the suggestions in an updated version of our manuscript.

# Response

**Published:** 2021-08-19

**Authors:** Johann-Mattis List

**Affiliations:** University of Passau, Germany

Dear Pui Yiu Szeto, we have now had time to revise the manuscript and submitted a new version. Below, we list your suggestions to improve the study and add our answers.

- *Reading the abstract, one will normally expect that languages spoken in Southeast Asian countries like Vietnam, Cambodia, Thailand, and Laos are considered in this study. However, all the languages involved are actually spoken in Southern China.*
  We have made sure to be more precise in the new version, specifying Southern China.
- *Given the considerable structural differences between Tai-Kadai languages spoken inside and outside China due to varying degrees of Sinitic influence (Szeto and Yurayong [forthcoming]), it will be interesting to incorporate at least a couple of Tai-Kadai languages spoken outside China to see whether they display a remarkably different pattern of lexical borrowing.*
  We now mention the idea to compare languages inside and outside of China as a potential case study in our conclusion.
- *I suggest using the Great Dictionary of Modern Chinese Dialects (Li 2002) as the sole source of Chinese dialect data. Li (2002) is a compendium of dictionaries for 42 Chinese dialects following a uniform format. This can surely improve the accuracy and coverage rate of the Chinese dialect data.*
  We added a paragraph in the methods section, in which we critically assess the potential of including the dictionary series by Lǐ (2002) and conclude that due to the way the data was gathered, it would unfortunately be very difficult (if not impossible) to extract the information in the formats needed for our work.
